# Workshop on Research Issues in Aluminum Toxicity

**DOI:** 10.1155/MBD.1995.245

**Published:** 1995


					WORKSHOP ON RESEARCH ISSUES
ALUMINUM TOXICITY
IN
Saturday, July 8, 1995
Vancouver Renaissance Hotel
1133 West Hastings Street, Vancouver, British Columbia
This workshop will differ from other conferences on aluminum in that the participants will identify and
discuss major unresolved/contrcversial issues related to aluminum that warrant future research and
suggest research approaches. The key participants addressing each issue will prepare a written
summary addressing their issue in advance of the workshop for presentation to the entire
delegation on.July 8. Summaries will be published in the Journal of Toxicology and Environmental
Health after the conference available as an issue of this journal to workshop registrants.
ISSUES TO BE DISCUSSED:
1. What are the status and future concerns of clinical and environmental aluminum toxicology?
2. What is the relevance and importance of aluminum chemistry, including biological speciation, to
the toxicity of aluminum?
3. What are the unresolved issues of aluminum toxicokinetics, including absorption, distribution and
blood-brain barrier permeation that need to be resolved?
4. What do we know and what do we need to know about the effects of aluminum during
developmental periods?
5. Can the mechanisms of aluminum-induced neurotoxicity be integrated into a unified scheme?
What is that scheme?
6. Can the controversy of the role of aluminum in Alzheimers disease be resolved? If so, how?
7. Aluminum biological standards: What are the needs?
8. How does aluminum exposure affect bone formation and remodeling, hematopoiesis, and renal
function?
9. Do we have adequate chelators or other methods to treat aluminum toxicity?
ORGANIZING COMMITTEE:
Robert A. Yokel, Ph.D.
University of Kentucky
John Savory, Ph.D.
University of Virginia
Mari S. Golub, Ph.D.
University of California-Davis
Chris Orvig, Ph.D.
University of British Columbia
Barbara F. Brandt, Ph.D.
University of Kentucky
KEY PARTICIPANTS:
Kenneth Abreo, M.D.
Peter Ackrill, M.D.
Allen C. Alfrey, M D.
Guy Beerthon, Ph.D.
J. Derek Birchall, O.B.E.
Karel Blaha, Ph.D.
Ellen BIIrgess, M.D.
Jorge U. Cannata, Ph.D.
Miroslav Cikrt Ph.D.
J. Philip Day, Ph.D.
Jose L. Domingo, Ph.D.
William D. Ehhmann, Ph.D.
Louisiana State Univ. Medical Center
University Hospital of South Manchester
University of Colorado
INSERM
University of Keele
Czech Ministry of Environment
University of Calgary
Hospital General Asturias
National Institute of Public Health, Czech Repllblic
University of Manchester
University of Barcelona
University of Kentucky
245
Christopher Exley, Ph.D.
Trond Peder Flaten, Ph.D.
William F. Forbes Ph.D.
Ralph M Garruto, Ph.D.
Mari S. Golub, Ph.D.
William G. Goodman, M.D.
Janet L. Greger, Ph .D.
Wesley R. Harris, Ph.D.
Elizabeth H. Jeffery, Ph.D.
Jayant G. Joshi, PhD.
Tamas Kiss, Ph.D.
Theo Kruck, Ph.D.
Mark A. Lovell, Ph.D.
William R. Markesbery, M.D.
R. Bruce Martin, Ph.D.
Sri Melethil, Ph.D.
William R. Mundy, Ph.D.
Chris Orvig, Ph.D.
John Savory, Ph.D.
Timothy J. Shafer ,Ph.D
Michael J. Strong, M D.
Carol R. Swyt, Ph.D.
Ikuro Wakayama, M.D.
David R. Williams, Ph D.
Henry M. Wisniewski, M.D., Ph.D.
Robert A. Yokel, Ph.D.
Khalequz Zamam, Ph.D.
Paolo Zatta, Ph.D.
University of Keele
University of Trodheim
Canadian Center of Health Info.
NIH
University of California, Davis
University of California-Los Angeles
University of Wisconsin-Madison
University of Missouri-St. Louis
University of Illinois-Champaign
University of Tennessee
Lajos Kossuth University
University of Toronto
University of Kentucky
University of Kentucky
University of Virginia
University of Missouri-Kansas City
US EPA
University of British Columbia
University of Virginia
US EPA
University Hospital, University of Western Ontario
NIH/BEIP
Wakayama Medical College
University of Wales
Institute for Basic Research in Dev Disab
University of Kentucky
University of Nevada
University of Padova
Registration Fee: $75.00 US* (includes breakfast and lunch)
*Discount with joint registration to COMTOX
Vancouver Renaissance Hotel
1 133 W. Hastings Street
Phone: (604)689-92 11
Room rate: $145.00 cdn ($105 US approx)
Rooms will be held until June 6, 1995
Please indicate you are part of the Aluminum Workshop program when making reservations
For more information on this workshop, contact:
The University of Kentucky
Continuing Pharmacy Education
465 E. High Street, Suite 204
Lexington, KY 40507
Phone" (606)257-7719 Fax: (606)323-2437
246

				

